# Pellicle Modification with Casein and Mucin Does Not Promote In Vitro Bacterial Biofilm Formation

**DOI:** 10.3290/j.ohpd.a43351

**Published:** 2020-07-04

**Authors:** Thiago Saads Carvalho, Judith Elisa Halter, Dea Muçolli, Adrian Lussi, Sigrun Eick, Tommy Baumann

**Affiliations:** a Senior Research Associate and Senior Lecturer, Department of Restorative, Preventive and Pediatric Dentistry, School of Dental Medicine, University of Bern, Bern, Switzerland. Experimental design, performed the statistical evaluation, wrote the manuscript.; b Student, Department of Restorative, Preventive and Pediatric Dentistry/Department of Periodontology, School of Dental Medicine, University of Bern, Bern, Switzerland. Performed the experiments and proofread the manuscript.; c Student, Department of Restorative, Preventive and Pediatric Dentistry/Department of Periodontology, School of Dental Medicine, University of Bern, Bern, Switzerland. Performed the experiments and proofread the manuscript.; d Professor, Department of Restorative, Preventive and Pediatric Dentistry, School of Dental Medicine, University of Bern, Bern, Switzerland. Critically reviewed the manuscript.; e Professor, Department of Periodontology, School of Dental Medicine, University of Bern, Bern, Switzerland. Experimental design, performed the statistical evaluation, critically reviewed the manuscript.; f Research Associate, Department of Restorative, Preventive and Pediatric Dentistry, School of Dental Medicine, University of Bern, Bern, Switzerland. Experimental design, critically reviewed the manuscript.

**Keywords:** biofilms, casein, demineralisation, enamel, mucin, salivary pellicle, salivary pellicle modification

## Abstract

**Purpose:**

During biofilm formation, bacterial species do not attach directly onto the enamel surface, but rather onto the salivary pellicle. Salivary pellicle modification with casein and mucin can hinder erosive demineralisation of the enamel, but it should also not promote bacterial adhesion. The aim of our study was to assess whether salivary pellicle modification with casein, or mucin, or a mixture of both proteins (casein and mucin) influence bacterial adhesion, biofilm diversity, metabolism and composition, or enamel demineralisation, after incubation in: (a) a single bacterial model; (b) a five-species biofilm model; or (c) biofilm reformation using the five-species biofilm model after removal of initial biofilm with toothbrushing.

**Materials and Methods:**

Enamel specimens were prepared from human molars. Whole-mouth stimulated human saliva was used for pellicle formation. Four pellicle modification groups were established: control (non-modified pellicle); casein – modified with 0.5% casein; mucin – modified with 0.5% mucin; casein and mucin – modified with 0.5% casein and 0.5% mucin. Bacterial adhesion, biofilm diversity, metabolic activity, biofilm mass, and demineralisation (surface hardness) of enamel were assessed after incubation in bacterial broths after 6 h or 24 h.

**Results:**

After 24 h incubation in the five-species biofilm model, the mucin group presented significantly lower biofilm mass than the control (p = 0.028) and the casein and mucin (p = 0.030) groups. No other differences between the groups were observed in any of the other experimental procedures.

**Conclusion:**

Pellicle modification with casein and mucin does not promote in vitro bacterial biofilm formation.

Dental caries is a major global problem, and has been the most prevalent health condition throughout the world.^12^ It is caused by acids generated by bacterial fermentation, when different bacterial species form a biofilm on the surface of the teeth. Bacterial deposition begins with the initial colonisation from streptococci and *Actinomycetes* spp. These microorganisms, however, do not attach directly onto the enamel surface, but rather onto the salivary pellicle.^16^

The salivary pellicle is a protein-rich layer that coats the surface of the teeth.^14^ Strong interactions between some salivary proteins and the tooth surface promote the early protein deposits, which later undergo further modulation from protein–protein interactions, culminating in a salivary pellicle comprising proline-rich proteins (PRPs), mucins (more specifically the mucin MUC5B), amylase, albumin, statherin, among others.^22^ These proteins contain receptors for bacteria that allow the interactions between the salivary pellicle and the bacteria, initiating the biofilm accumulation.^8^ In consequence, the pellicle directly influences the initial microbial colonisation,^16^ and modifying the salivary pellicle by altering its protein composition can have a direct impact on the adhesion of bacteria, which, in turn, could ultimately modify the quality of the biofilm.

Salivary pellicle modification has already been attempted with different proteins, including mucin and casein.^4,5,20,21^ Mucins are glycoproteins that constitute the major structural component of mucus; while casein is a protein commonly found in dairy products. Its main function in milk is to stabilise calcium phosphate by forming micelles. It can also bind directly to hydroxyapatite, forming a thin layer that restricts the access of the acid to the surface of the crystals, thus inhibiting the dissolution of calcium and phosphate.^1^ Casein can also incorporate into the salivary pellicle,^21^ and, in combination with mucin, it can modify the salivary pellicle improving its erosion-inhibiting properties.^4^

In addition to hindering erosive demineralisation, salivary pellicle modification with casein and mucin can also significantly reduce the adhesion of the early colonisers, as described by Cheaib, Rakmathulina, Lussi and Eick.^5^ However, the authors^5^ only tested single bacterial species of early colonisers with incubation time of up to 2 h, whereas a multispecies biofilm model with several cariogenic species and a longer follow-up period and incubation times that include a toothbrushing challenge would be more representative of the clinical situation. Therefore, the aims of this study were to investigate whether the pellicle modification with casein, or mucin, or a mixture of both proteins (casein and mucin) would have an influence on: (1) the bacterial adhesion using *Streptococcus gordonii* as a single bacterial species for 30 min; (2) the formation, metabolism and composition of a cariogenic biofilm consisting of five species, as well as cariogenic demineralisation of the enamel after 24 h incubation; (3) reformation of a cariogenic biofilm over 24 h and cariogenic demineralisation of enamel, using a five-species biofilm model formed on the enamel after mechanical removal (toothbrushing) of an initial biofilm.

## Materials and Methods

### Preparation of Human Enamel Specimens

Teeth were taken from a pooled biobank. Thus, they were categorised as ‘irreversibly anonymised’ and no previous ethical approval from the local ethics committee (Kantonale Ethikkommission: KEK) was necessary for this study. The experiment was made in accordance with approved guidelines and regulations, and the patients provided oral consent for the use of their teeth in research. The teeth had been extracted by dental practitioners in Switzerland (no water fluoridation) and were stored in 2% chloramine T trihydrate solution.

For this study, around 150 enamel specimens were prepared from human molars. The crowns were separated from the roots and then bisected into buccal and oral halves using an IsoMet Low Speed Saw (Buehler, Düsseldorf, Germany). The buccal and oral halves were then embedded in resin Paladur (Kulzer, Hanau, Germany), ground and polished under water cooling (Struers Knuth-Rotor 2 and LaboPol 21, Rodovre, Denmark) and using silicon carbide papers with decreasing grain sizes of 18, 10 and 5 µm. Between every polishing or grinding step, the specimens were washed with tap water and ultrasonicated for 1 min.

The grinding procedure removed the outermost 200 µm of enamel and resulted in smooth, polished and planar parallel specimens. The enamel specimens were stored in a mineral solution (1.5 mmol/L CaCl_2_, 1.0 mmol/L KH_2_PO_4_, 50 mmol/L NaCl, pH 7.0)^28^ until the time of the experiment. Immediately prior to the experimental procedures, the initial surface microhardness (SMH) of all enamel specimens was measured.

### Collection of Whole-Mouth Stimulated Human Saliva

The saliva was collected and immediately pooled. And, as being categorised as ‘irreversibly anonymised’, again, previous ethical approval from the local ethics committee was not necessary. The saliva donors gave their informed oral consent to use the saliva for research purposes.

Whole-mouth stimulated human saliva was collected from adult, healthy volunteers who chewed on paraffin tablets for 10 min. The saliva was collected into chilled vials and pooled directly after collection. In order to remove the debris, the saliva was centrifuged for 20 min at 4°C and 4000 g. The supernatant was collected, sterilised through exposure to ultraviolet (UV) radiation for 30 min, and the sterile saliva was divided into aliquots and stored at –80°C. Sterility of the saliva was tested by cultivation, and immediately prior to the experiment, the aliquots of saliva were defrosted and used.

### Pellicle Formation/Modification and Experimental Groups

For pellicle formation, 50 µl of saliva was placed onto each enamel specimen for 2 h. For pellicle modification, the enamel specimens were then incubated in one of four protein solutions. The experimental groups were as follows:

1. Control group: non-modified pellicle layer (only deionised water was used);
2. Casein group: pellicle layer was modified with a 0.5% casein solution (casein from bovine milk; Sigma-Aldrich, Saint Louis, MO, USA);
3. Mucin group: pellicle layer was modified with a 0.5% mucin solution (mucin from porcine stomach; Sigma-Aldrich);
4. Casein and mucin group: pellicle layer was modified with a solution containing 0.5% casein and 0.5% mucin.

The solutions were prepared by dissolving the protein in deionised water. The pH of the water was raised with potassium hydroxide (KOH) to 11.5, and the water was heated to 40°C. When the protein was dissolved, the solution was cooled to room temperature and the pH adjusted to 7 (with HCl). Incubation in each of these solutions was made at room temperature, for 30 min, under sterile conditions. Immediately after pellicle modification, the specimens were subjected to bacterial adhesion assays.

### Surface Microhardness Measurement

Surface microhardness (SMH) measurements were performed using a Knoop diamond under a load of 50 g and a dwell time of 10 s (UHL VMHT Microhardness Tester, UHL Technischer Mikroskopie, Aßlar, Germany). For each SMH measurement, six indentations were made at intervals of 50 μm, and the average of these six indentations was considered as the SMH value for the specimen. For statistical analyses, relative SMH (rSMH) was calculated using the formula: rSMH = (SMH_final_ / SMH_initial_) × 100.

### Bacterial Strains

The following bacteria strains were used in this study: *Streptococcus gordonii* ATCC 10558, *S. mutans* ATCC 25175, *S. sobrinus* ATCC 12104, *Lactobacillus acidophilus* ATCC 11975 and *Actinomyces naeslundii* ATCC 12104. Prior to the experiment, the strains were passaged for 24 h on tryptic soy agar (TSA) plates (Oxoid, Basingstoke, UK) with 5% sheep blood, 10% CO_2_, at 37°C.

### Bacterial Adhesion Assays and Cariogenic Demineralisation

This experiment was carried out in three parts: (1) analysis of bacterial adhesion using a single bacterial species; (2) analysis of bacterial adhesion and cariogenic demineralisation using a five-species biofilm; (3) analysis of further bacterial adhesion and cariogenic demineralisation after mechanical removal of initial biofilm with a toothbrush.

#### Analysis of bacterial adhesion using a single bacterial species

For this experimental setup, *S. gordonii* was used as the single bacterial species. A total of 32 enamel specimens were distributed into the four pellicle modification experimental groups (n = 8). After pellicle formation and modification, the specimens were contaminated with *S. gordonii*, in a bacterial suspension (OD60 0= 0.1; equivalent to 108 bacteria/ml) in Dulbecco’s modified Eagle’s medium (DMEM; Gibco, Invitrogen). The specimens were incubated at 37°C with 10% CO_2_ for 30 min.

After 30 min, bacterial adhesion to the enamel specimens was quantified by determining bacterial counts (colony forming units (cfu)). This was made by washing the specimens once with 0.9% w/v NaCl solution to remove non-adhered bacteria, then the enamel surface was scraped with a cotton swab to remove the adhered biofilm, and the swabs were then placed and dispersed in 1 ml 0.9% (w/v) NaCl solution. This suspension was then plated onto TSA with 5% sheep blood. After an incubation at 10% CO_2_, and 37°C, for 48 h, the cfu were then counted.

#### Analysis of bacterial adhesion and cariogenic demineralisation using a five-species biofilm

A total of 80 enamel specimens were used: 32 specimens (8 per group) were used to verify bacterial adhesion after 6 h incubation; 48 specimens (12 per group) were used for biofilm formation and cariogenic demineralisation after 24 h incubation.

Initially, enamel surface microhardness was measured in the 48 specimens used for the 24 h assay (SMH_initial_).

All 80 specimens were then submitted to pellicle formation and modification, then contaminated with a five-species bacterial suspension consisting of a mixture of the following five bacterial species: suspensions of *S. gordonii*, *S. mutans*, *S. sobrinus*, *L. acidophilus* and *A. naeslundii* (McFarland 4 in 0.9% w/v NaCl) were mixed in a ratio of 1:1:2:2:3, before adding in a ratio 1:9 to brain-heart infusion (BHI) broth (BioMérieux, Marcy l’Etoile, France) with 5% sucrose and incubated for 6 h at 37°C with 10% CO_2_.

After the 6 h of incubation with 10% CO_2_ at 37°C, the 32 specimens used for the bacterial adhesion were removed from the experiment and treated as described above. The cfu counts were recorded considering the different bacterial strains. Also, the suspension containing the biofilm was used to analyse the metabolic activity and the biofilm mass, as described in previous studies.^13,18,19^ For metabolic activity, Alamar blue (alamarBlue reagent, Thermo Fisher Scientific, Waltham, MA, USA) was used. This is a resazurin-based solution, from which 5 µl was mixed with 100 µl of nutrient media and added to the suspended biofilm. The mixture was incubated for 1 h at 37°C, and then analysed with an absorbance microplate reader (ELx808, Biotek Instruments, Winooski, VT, USA). Differences of absorbances at 600 nm to 570 nm wavelengths were calculated. For biofilm mass, the suspended biofilm was fixed at 60°C for 1 h, then stained with 0.06% (w/v) crystal violet (Sigma-Aldrich Chemie) for 10 min. The staining was then quantified using the absorbance microplate reader at 600 nm wavelength.

At 6 h, the other 48 specimens used for bacterial adhesion and cariogenic demineralisation were removed from the BHI broth with 5% sucrose medium and placed in a BHI containing phosphate buffer (0.021 mol/L KH_2_PO_4_; 0.016 mol/L Na_2_HPO_4_). The specimens were left in incubation for a further 18 h at 37°C with 10% CO_2_, thus totalling 24 h incubation. Afterwards, the bacterial counts, metabolic activity and mass of the biofilm were analysed as described previously. Enamel demineralisation was also analysed on these enamel specimens by measuring the final surface microhardness (SMH_final_).

#### Biofilm reformation, analysis of and further bacterial adhesion, and cariogenic demineralisation after mechanical removal of initial biofilm with toothbrush

For this assay, only two experimental groups were of interest: the pellicle-modified group with 0.5% aq. mucin/0.5% aq. casein (Group 3), and the non-modified-pellicle (Group 4). A total of 16 enamel specimens were distributed into these two experimental groups (8 specimens/group). Initially, enamel surface microhardness (SMH_initial_) was measured for all specimens. Then enamel specimens were subjected to pellicle formation and modification, and later contaminated with the five-species bacterial suspension, as described in part 2. The specimens were incubated in the suspension for a total of 24 h (6 h in BHI with sucrose and 18 h in BHI with added buffer), the biofilm was mechanically removed by toothbrushing. For that, the enamel specimens were individually brushed with soft toothbrushes, with a force of 1.5 ± 0.05 N, 1 stroke/s, for a total of 10 s.

After brushing, the specimens once again underwent a short dipping into 0.9% NaCl, pellicle formation and modification. For further biofilm formation, the specimens were again contaminated with the five-species bacterial suspension and incubated for another 24 h (6 h in BHI with sucrose and 18 h in BHI with buffer). Finally, cfu counts, metabolic activity, biofilm mass and cariogenic demineralisation were assessed as before.

### Statistical Analyses

Results for each part of the study were analysed separately. For parts 1 and 2, one-way analysis of variance (ANOVA) with post-hoc least statistically significant difference (LSD) tests were carried out for total cfu counts (log10), amount (%) of cariogenic streptococci (*S. mutans*, *S. Sobrinus*) and *L. acidophilus*, metabolic activity of the biofilm, biofilm mass and enamel surface microhardness. For part 3, independent samples t tests were carried out for the same variables.

## Results

### Analysis of Bacterial Adhesion Using a Single Bacterial Species

**Fig 1:**
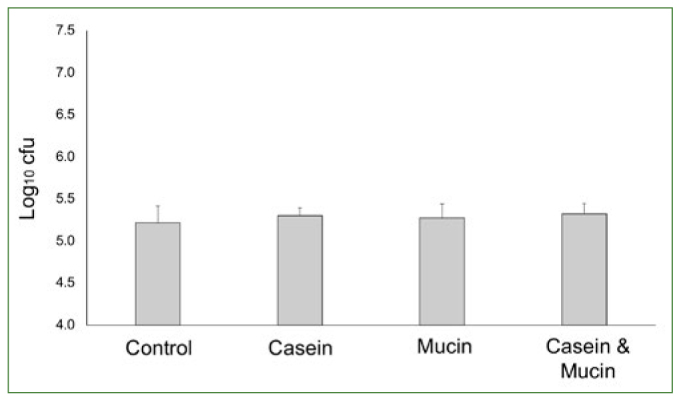
Bar chart showing the mean and standard deviation of colony forming units (Log10 cfu) using a single bacterial species (*S. gordonii*) model after 30 min incubation. NOTE: cfu = colony forming units (Log10 cfu); no statistical differences between the groups (p = 0.540); ANOVA with post-hoc LSD tests; total of eight cases per group in the analysis.

When only *S. gordonii* ATCC 10558 was used in the bacterial adhesion assay, no statistically significant difference (p = 0.540) was found in the number of cfu in the different pellicle groups (Fig 1).

#### Analysis of bacterial adhesion and cariogenic demineralisation using a five-species biofilm

**Fig 2:**
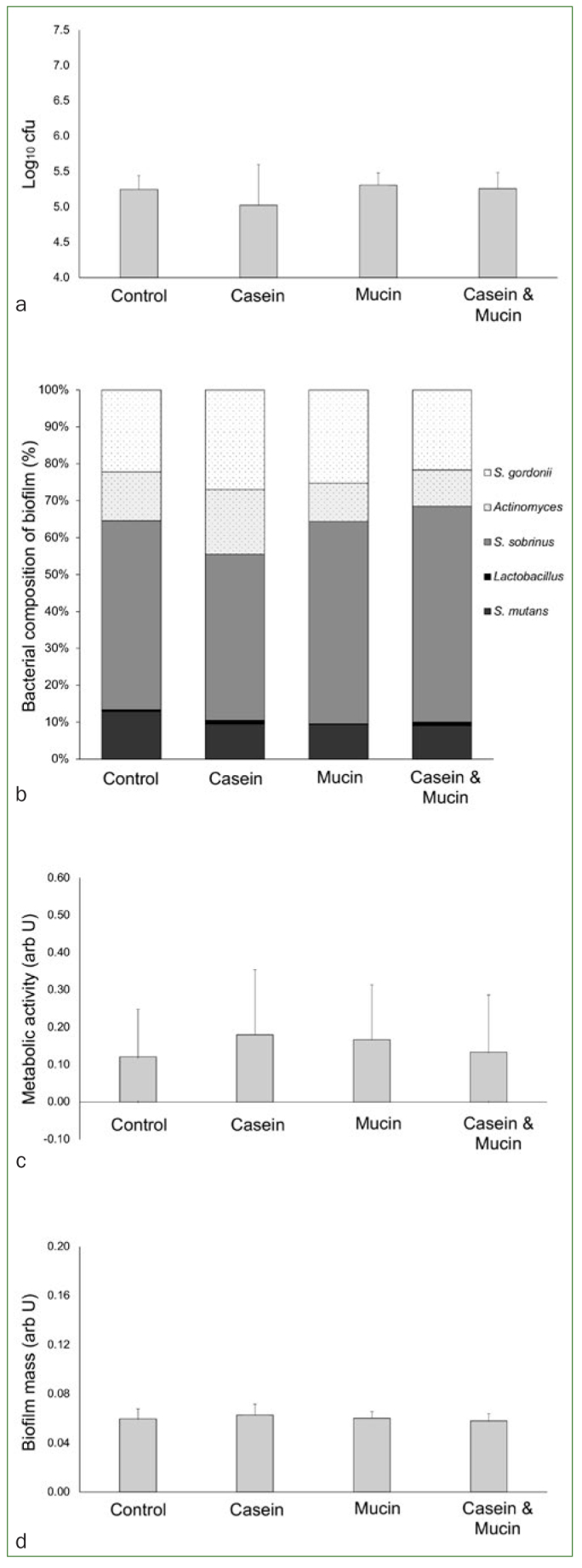
Bacterial adhesion using a five-species biofilm after 6 h incubation in cariogenic medium. (a) Bar chart (mean and standard deviation (SD)) showing the count of colony forming units (Log10 cfu); (b) bar diagram with the proportion of different bacterial species in the biofilm (darker subdivisions indicate cariogenic microorganisms; lighter (pattern) subdivisions indicate non-cariogenic microorganisms); (c) bar chart (mean and SD) showing the metabolic activity of the biofilm; (d) bar chart (mean and SD) showing biofilm mass. Note: no statistical difference between the groups was observed (p >0.05) in any of the assays; ANOVA with post-hoc LSD tests; total of eight cases per group in the analyses.

**Fig 3:**
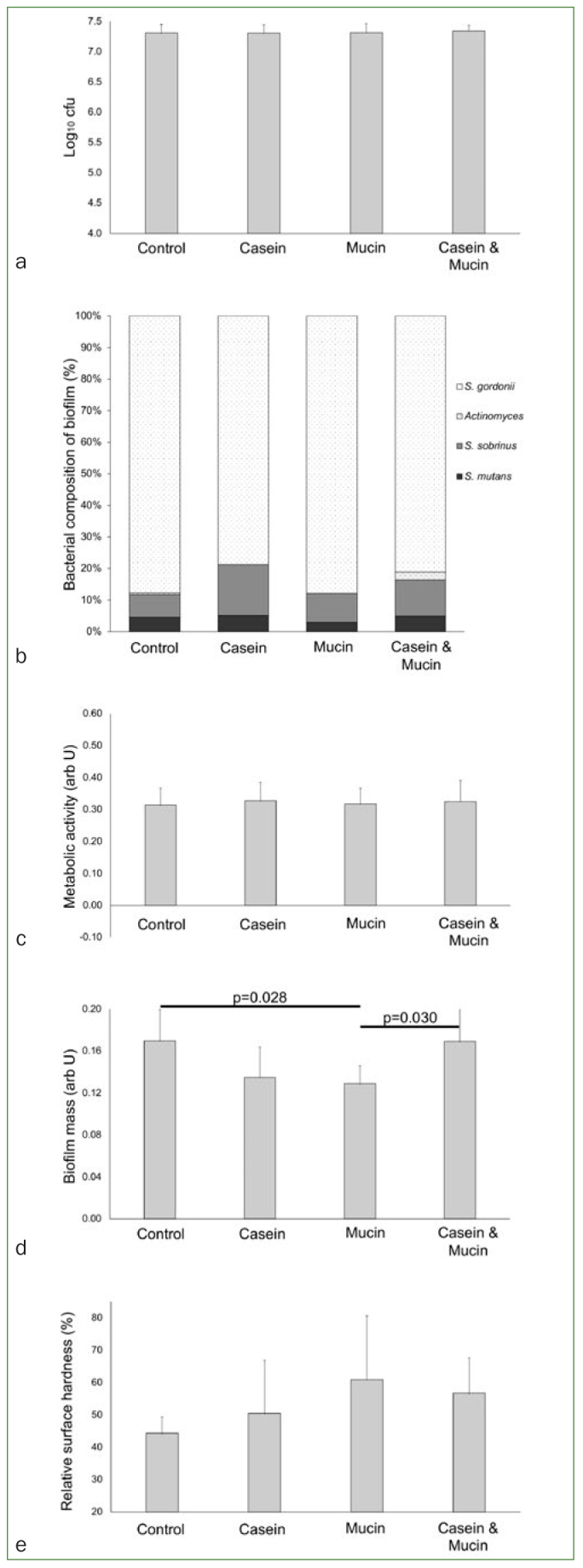
Bacterial adhesion and cariogenic demineralisation using a five-species biofilm after 24 h incubation (6 h in cariogenic medium, and 18 h in non-cariogenic medium). (a) Bar chart (mean and SD) showing the count of colony forming units (cfu); (b) bar diagram with the proportion of different bacterial species in the biofilm (Darker subdivisions indicate cariogenic microorganisms; lighter (pattern) subdivisions indicate non-cariogenic microorganisms); (c) bar chart (mean and SD) showing the metabolic activity of the biofilm; (d) bar chart (mean and SD) showing the biofilm mass; (e) bar chart (mean and SD) showing the enamel surface microhardness (taller bars indicate less change in microhardness, therefore less demineralisation). NOTE: statistical differences were observed in biofilm mass (D), where pellicle modified with mucin presented significantly lower biofilm mass than the non-modified pellicle (p = 0.028) or the pellicle modified with the mixture of casein and mucin (p = 0.030). No other statistical difference was observed (p >0.05) in the other assays; ANOVA with post-hoc LSD tests; total of 12 cases per group in the analyses of figures A, B and E; total of eight cases per group in the analyses of figures C and D.

The analysis using a five-species biofilm was carried out after 6 h incubation (Fig 2) in medium containing 5% sucrose, and 24 h (Fig 3) incubation (6 h in medium with 5% sucrose, and 18 h in medium with buffer). After 6 h incubation in cariogenic medium, bacterial adhesion to the enamel specimens did not differ between the groups (Fig 2a; p = 0.352), and the values were within a similar range as those observed in the single bacterial species assay (Fig 1a). The adhered bacteria were different species (Fig 2b), and over 50% of them were cariogenic species (*S. sobrinus*, *L. acidophilus*, and *S. mutans*), but no difference was observed in the number of cariogenic species between the groups (p = 0.445). Similarly, there were no statistically significant differences between the groups in the metabolic activity of the biofilm (Fig 2c; p = 0.847), in the biofilm mass (Fig 2d; p = 0.622).

After 24 h incubation, a considerably larger number of cfu was observed, but there was no statistically significant difference between the groups (Fig 3a; p = 0.900). Analysis of bacterial composition of biofilms showed that cariogenic species of the adhered bacteria ranged from 12% to 21% (Fig 3b), with no statistically significant difference between the groups (p = 0.562). Also, no statistically significant difference was observed in the metabolic activity of the biofilm (Fig 3c; p = 0.965). Statistical differences were observed in the biofilm mass (Fig 3d; p = 0.044), where the biofilm formed on pellicle modified with mucin presented significantly lower mass than the non-modified pellicle (p = 0.028) or on the pellicle modified with the mixture of casein and mucin (p = 0.030). Cariogenic demineralisation of the enamel was observed in all groups (Fig 3e), enamel surface hardness decreased only by 39% in the mucin group, and by 56% in the control group, but the differences did not reach statistical significance (p = 0.281).

#### Analysis of biofilm reformation and cariogenic demineralisation after mechanical removal of initial biofilm with toothbrush

**Fig 4:**
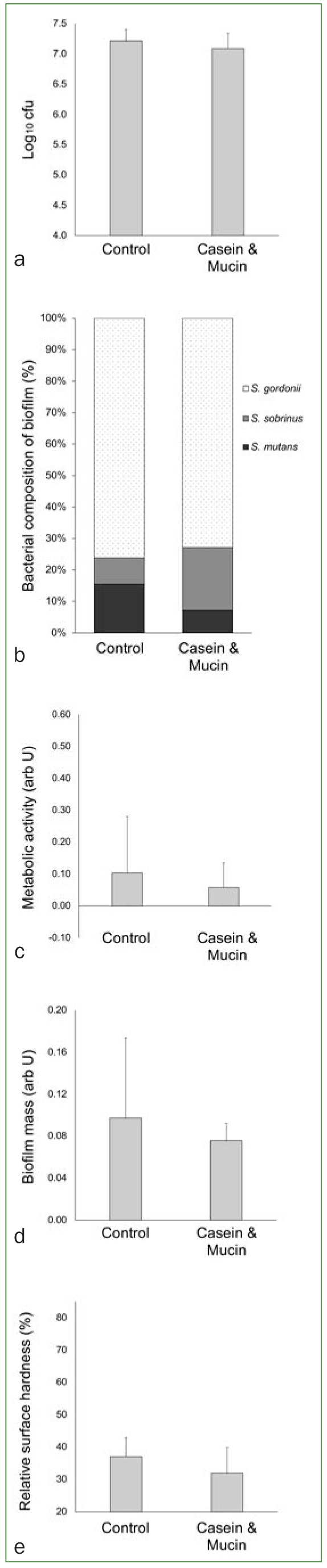
Further bacterial adhesion and cariogenic demineralisation after mechanical removal of an initial biofilm with toothbrush. (a) Bar chart (mean and SD) showing the count of colony forming units (cfu); (b) bar diagram with the proportion of different bacterial species in the biofilm (darker subdivisions indicate cariogenic microorganisms; lighter (pattern) subdivisions indicate non-cariogenic microorganisms); (c) bar chart (mean and SD) showing the metabolic activity of the biofilm; (d) bar chart (mean and SD) showing the biofilm mass; (e) bar chart (mean and SD) showing the enamel surface microhardness (taller bars indicate less change in microhardness, therefore less demineralisation). Note: no statistical difference between the groups was observed (p >0.05) in any of the assays; independent t-test analyses; total eight cases per group in the analyses.

Further bacterial biofilm counts after a mechanical removal of the initial biofilm showed no statistically significant differences between the two tested groups (Fig 4a; p = 0.271). Bacterial composition analyses showed that cariogenic streptococci and *L. acidophilus* of the adhered bacteria ranged between 24% and 27% for the control and the casein/mucin groups, respectively (Fig 4b), with no statistically significant differences between them (p = 0.800). The casein and mucin group by trend presented lower numerical values than the control group for the metabolic activity of the biofilm (Fig 4c) and for the biofilm mass (Fig 4d), but these differences failed statistical significance (p = 0.518, and p = 0.443 for the metabolic activity and the biofilm mass, respectively). Similarly, no differences were observed in the cariogenic demineralisation between the groups, here surface hardness loss had the trend to be higher in the casein and mucin group than in the controls (Fig 4e; p = 0.173).

## Discussion

This series of experiments aimed at investigating whether the pellicle modification with casein, or mucin, or a mixture of both proteins (casein and mucin) would have an influence on bacterial adhesion, initial biofilm formation, biofilm metabolism and composition. Previous studies have shown that pellicle modification can have a positive effect on erosion prevention.^4,10,27^ However, since bacterial adhesion does not occur directly on the enamel surface, but rather on the salivary pellicle, it follows that alterations to the pellicle will also influence bacterial adhesion. Some studies have shown that pellicle modification with tea,^9^ tannic acid,^10^ or mucin and casein,^5^ all had positive effect in hindering bacterial colonisation onto enamel specimens, but a side effect of the pellicle modification could be the promotion of bacterial adhesion and the subsequent demineralisation. So, further studies on this topic are still necessary, especially using multispecies biofilm.

To the best of our knowledge, this is the first study that verifies the effect of pellicle modification with casein and mucin using a multispecies biofilm formed on human enamel. The vast majority of other studies have either used hydroxyapatite discs and bovine enamel as substrates for pellicle formation, or they have only used single bacterial species in their experimental models. This could lead to non-representative results. On the one hand, different kinds of substrates will lead to different kinds of salivary pellicles,^3,23,29^ so it is crucial to use human enamel and human saliva when dealing with pellicle studies. On the other hand, experimental models using single bacterial species do not mirror the multispecies biofilm consortium established in in vivo situations. Therefore, ideally multispecies biofilm models should be used. Our study, therefore, used human enamel, human saliva and different bacterial adhesion models, comprising of three parts: (1) analysis of early bacterial adhesion using a single bacterial species; (2) analysis of biofilm formation and cariogenic demineralisation by using a five-species bacterial mixture for up to 24 h; (3) analysis of biofilm formation and cariogenic demineralisation after mechanical removal of initial biofilm with toothbrush.

The idea of using mucin and casein to modify the salivary pellicle was based on the positive effects of these proteins on tooth demineralisation. Mucin is a principal component of saliva, making up 20–30% of the total protein content in unstimulated whole saliva. Salivary mucins are mainly distinguished into two main types: MUC5B, a high molecular weight mucin, and MUC7, a low-molecular weight mucin, both containing covalently-bonded carbohydrate side chains that provide the molecules an elongated form.^26^ These side chains also provide the molecules with hydrophilic properties, which could also serve as potential receptors for microorganisms. The low-molecular weight MUC7 has been shown to bind to several oral microorganisms, including *S. mutans*, whereas the high molecular weight MUC5B binds, comparatively, to fewer oral microorganisms.^26^ In the present experiment, we used pig gastric mucin, which is roughly twice the size of the human salivary mucin MUC5B 4, and could imply a greater number of carbohydrate side chains available as potential receptors for microorganisms. However, pig gastric mucin has shown no increase in the adhesion of oral microorganisms: *S. gordonii*, *A. odontolyticus*, *S. oralis,*^5^ or *S. mutans.*^7^ So it was expected that the group of pellicle modified with mucin or the mixture of casein and mucin should not increase bacterial adhesion. This was the case in our investigation, where no increase in biofilm formation was observed in any of the pellicle modification groups. Moreover, biofilm mass could be influenced by the bacteria count, so we calculated the biofilm mass with respect to cfu counts. Further analyses showed that, despite the statistically significant decrease in mass of the biofilm formed on the pellicle modified with mucin (Fig 3d) after 24 h incubation in a five-species biofilm, no differences were observed in the mass when accounting for cfu counts. Similarly, all further analyses of this biofilm/cfu mass showed no differences between any of the groups.

Casein is the most predominant phosphoprotein found in milk, making up ~80% of the total protein content.^2^ In bovine milk, casein is mainly distinguished into four main types: _aS1_-casein amounts to 40% of the total casein fraction; _αS2_-casein comprises 10%; β-casein represents 45%; and k-casein makes up 5% of the total casein fraction.^6^ Previous studies have shown that these different kinds of caseins have different effects on oral microorganisms. Vacca-Smith, Van Wuyckhuyse, Tabak and Bowen^25^ observed that, when k-casein was incorporated onto pellicle-covered hydroxyapatite discs, it was able to inhibit adhesion of *S. mutans*, though α- and β-casein did not present the same antiadhesion outcome. Schupbach, Neeser, Golliard, Rouvet and Guggenheim^21^ used pellicle-covered bovine enamel, and observed a reduction in the adherence of *S. mutans* and *S. sobrinus* when casein was incorporated onto salivary pellicle. The authors further suggested that this inhibition was mediated not only by the k-casein, but also by the β-casein, since both structures are able to adsorb onto the pellicle and mask the receptors of salivary molecules for streptococci.^21^ In our study, we did not discriminate the different types of casein, but rather used whole casein from bovine milk, probably containing the ratios for the _α__S1_-, _α__S2_-, β-, and k-casein described above. Since similar amounts of α-, β- and k-casein bind to the pellicle,^25^ we speculate that the α-caseins ‘competed’ with the β- and k-casein for binding sites to the pellicle. So, in our study, ~50% of the caseins incorporated to the modified pellicle were probably of the _α__S1_- and _α__S2_- types, which do not exert any effect on bacterial adhesion. In their in situ study, Vacca-Smith and Bowen^24^ observed significantly less bacteria adhesion after rinsing hydroxyapatite specimens with pure k-casein. The authors suggested that k-casein functions as an antiadhesion agent by either replacing the bacteria on the hydroxyapatite or by adhering to the hydroxyapatite faster than the bacteria.^24^ We can, therefore, speculate that pure k-casein would probably lead to a more evident antiadhesion effect. Moreover, the casein would probably have also interacted with the enamel surface,^1,24^ leading to less enamel demineralisation.

In part 1 of our study, we analysed the adhesion of *S. gordonii* using a single bacterial species model, and observed no statistically significant differences in the number of cfu between the different pellicle modification groups. Under similar conditions, Cheaib, Rakmathulina, Lussi and Eick^5^ observed that pellicle modification with either mucin, or casein or their mixture yielded a statistically significant reduction in *S. gordonii* adhesion. These contradictory results could either be due to the different modification times or the different mucin concentrations used in the two studies: Cheaib, Rakmathulina, Lussi and Eick^5^ used 0.5% casein and 0.27% mucin concentrations, and they modified the pellicle by incubating the specimens for 2 h in the modifying (protein) solutions; whereas we presently used 0.5% casein and 0.5% mucin concentrations, and we incubated our specimens for only 30 min. These differences were preferred due to methodological reasons and better initial results in other erosion-related experiments (unpublished results). These differences, on the other hand, could also have affected the amount of mucin and casein bound to the pellicle, hence the lack of differences in the adhesion of bacteria.

In part 2, using a five-species biofilm model, we analysed bacterial adhesion and metabolic activity at two different times: after 6 h incubation in cariogenic biofilm, and after 24 h incubation (6 h in cariogenic medium, and 18 h in non-cariogenic medium). After 6 h incubation in cariogenic medium, more than 50% of the bacteria in all groups were cariogenic streptococci and *L. acidophilus*, and after an additional 18 h in non-cariogenic medium, this number decreased to less than 30%, which underlines the role of buffer in the surrounding environment. This decrease, however, caused no effect on metabolic activity of the biofilm or on the biofilm mass considering cfu count.

In analysing the results of biofilm mass, one should not only attend to the possibility that bacterial count could influence the data, but also the pellicle itself. Since the method of crystal violet stains carbohydrates, it could also be suggested that glycoproteins in the pellicle could be stained, and thus slightly overestimate the results. However, when biofilm is formed on the pellicle, the number of bacteria is considerably high, so bacterial count is far more likely to influence the biofilm mass than the pellicle itself. Still, no statistically significant differences were observed in the biofilm mass results when considering the cfu counts.

## Conclusion

Although our study showed no apparent effect of the pellicle modification on the bacterial biofilm and enamel demineralisation, we speculate that k-casein could still lead to better results in a twofold manner: (1) the k-casein would be able to bind to the enamel surface, hindering the contact of the acid with the enamel and upholding less demineralisation 1; and (2) the k-casein would bind to the salivary pellicles forming micelle-like structures, promoting the adsorption of more mucin molecules onto the modified pellicle,^4,15^ which would act as a carrier of smaller proteins to this pellicle.^11^ Having only k-casein in the modified pellicle and on the enamel surface, instead of k- and k-casein, would, in turn, decrease bacterial adhesion.^24^ Furthermore, casein has the ability to stabilise calcium phosphate. Likewise, mucin has the ability to interact with calcium and fluoride in combination with fluoridated toothpastes, and enhance the diffusion of calcium into a demineralised enamel lesion.^17^ Perhaps the mixture of k-casein and mucin could also positively influence enamel remineralisation. So, taking all the above-mentioned points into consideration, further in situ/in vivo tests analysing pellicle modification with k-casein and salivary mucins may be justified.

In conclusion, our results suggest that pellicle modification with casein or mucin did not promote bacterial colonisation. So clinical use of casein and mucin may be a possible approach to enhance erosion prevention without promoting any cariogenic response.
